# LPS-primed CD11b^+^ leukocytes serve as an effective carrier of Shiga toxin 2 to cause hemolytic uremic syndrome in mice

**DOI:** 10.1038/s41598-018-22327-4

**Published:** 2018-03-05

**Authors:** Shuo Niu, John Paluszynski, Zhen Bian, Lei Shi, Koby Kidder, Yuan Liu

**Affiliations:** Program of Immunology & Molecular Cellular Biology, Department of Biology, Center for Diagnostics & Therapeutics, Center of Inflammation, Immunity and Infection, Georgia State University, Atlanta, GA 30302 USA

## Abstract

Shiga toxin (Stx)-induced hemolytic uremic syndrome (HUS) is a life-threatening complication associated with Stx-producing *Escherichia coli* infection. One critical barrier of understanding HUS is how Stx transports from infected intestine to kidney to cause HUS. Passive dissemination seems unlikely, while circulating blood cells have been debated to serve as the toxin carrier. Employing a murine model of Stx2-induced HUS with LPS priming (LPS-Stx2), we investigate how Stx causes HUS and identify possible toxin carrier. We show that peripheral white blood cells (WBC), but not other blood cells or cell-free plasma, carry Stx2 in LPS-Stx2-treated mice. The capability of WBC binding to Stx2 is confirmed in brief *ex vivo* Stx2 incubation, and adoptively transferring these Stx2-bound WBC into mice induces HUS. Cell separation further identifies a subpopulation in the CD11b^+^ myeloid leukocytes not the CD11b^−^ lymphocytes group act as the toxin carrier, which captures Stx2 upon exposure and delivers the toxin *in vivo*. Interestingly, LPS-induced inflammation significantly augments these leukocytes for binding to Stx2 and enhances HUS toxicity. Our results demonstrate that a specific fraction of circulating leukocytes carry Stx2 and cause HUS *in vivo*, and that LPS priming enhances the carrier capacity and aggravates organ damage.

## Introduction

Shiga toxin (Stx)-producing *E*. *coli* (STEC) are virulent food-borne pathogens that annually cause over 2 million acute illnesses worldwide, among which several thousands require hospitalization and hundreds succumb to death^1–4^. Severe bacteria-induced enteritis with extensive diarrhea, both watery and bloody, is the major manifestation of STEC infection; however, fatality sometimes is attributable to hemolytic uremic syndrome (HUS)^5–8^. HUS, characterized by hemolytic anemia, acute kidney failure (uremia) and thrombocytopenia, often occurs abruptly at the post-acute, or even the recovery stage of STEC-induced enteritis. HUS development is associated with the spread of Stx to target organs such as kidneys, where high affinity cell surface receptors such as globotriaosylceramide (Gb3) mediate Stx internalization, resulting in cellular toxicity^9–12^. Two types of toxins, Stx1 and Stx2, can be produced by STEC, with Stx2 possessing much higher potency of toxicity. No specific therapeutics are available for treatment of HUS. In fact, traditional antibiotics promote expression of the toxin from the lysogenized phage that typically carries the Shiga toxin (Stx) gene, thereby exacerbating HUS severity^13^. Another challenge for clinicians is the absence of clinical tests that might identify those STEC-infected patients at risk of developing HUS as opposed to recovering from enteritis without witnessing symptoms of infection in distal organs^14^.

One major conundrum regarding HUS is how Stx is delivered from STEC-infected intestines to the kidneys. Unbound Stx molecules have not been detected in the serum of HUS patients, arguing against the possibility of passive dissemination^15–17^. Instead, particular studies implicate circulating leukocytes as the carriers of Stx to distant organs^18,19^. There are reports of Stx directly binding to human neutrophils^18,20^. Moreover, the Toll-like receptor 4 (TLR4) ligand, LPS, interferes with Stx binding to neutrophils, suggesting that TLR4 is the receptor by which Stx binds to neutrophil^21,22^. However, other studies either failed to replicate Stx binding to human neutrophils or demonstrated that the binding was unspecific or weak^23,24^. An *in vitro* study in another species showed that Stx, when co-incubated with porcine peripheral leukocytes, does bind to leukocytes; however, this study failed to identify which leukocyte bound to the toxin^25^. Studies have also been inconsistent regarding whether Gb3 is the leukocyte receptor for Stx2. In mice, a study demonstrated that Stx2 binds to Gb3 and is then internalized by the neutrophil, presumably resulting in degradation of the Stx2^26^. In a human study, Stx binding to neutrophils was independent of Gb3^11,22^. In addition to these inconsistent findings, a recent study reported detecting Stx2 in plasma microvesicles that were derived from platelets, leukocytes and red blood cells^27^. It should be noted that all of the previous studies failed to demonstrate a critical factor for relevance to the disease: the carrier can deliver Stx2 to the kidneys where HUS is subsequently manifested.

In this study, we employ the LPS-Stx2 murine HUS model^28,29^ to further interrogate how Stx2 is carried to the kidneys where it causes HUS. Our data show that circulating CD11b^+^ myeloid leukocytes from LPS-primed mice are highly effective carriers of Stx2 *in vivo*. Brief exposure of these leukocytes to Stx2, even *ex vivo*, results in toxin binding and enables their capability to cause HUS *in vivo*. In addition, we demonstrated for the first time that LPS-induced systemic inflammation primes myeloid leukocytes to become highly capable Stx2 carriers. The LPS-primed leukocytes were able to cause severe kidney damage associated with HUS.

## Materials and Methods

### Murine LPS-Stx2 HUS model

LPS-primed Stx2-induced HUS (LPS-Stx2 HUS) in mice^28^ was established for the study. C57BL/6 mice (6–10 weeks old, 18–22 g) purchased from the Jackson Laboratory were housed in a pathogen-free animal facility. All experiments using animals and procedures of animal care and handling were carried out in strict accordance with the recommendations in the Guide for the Care and Use of Laboratory Animals of NIH. The protocols for animal studies were approved by the Institutional Animal Care and Use Committee (IACUC) of Georgia State University (Atlanta, GA). Stx2 was obtained from the Phoenix Laboratory at Tufts Medical Center (Boston, MA). To induce HUS in mice, one dose of LPS (300μg/kg, Sigma-Aldrich) was first administered intraperitoneally (i.p.) to prime the mice. After 24 h, Stx2 was given *i*.*p*. at the dose of 500 ng/kg (~2 × LD50)^30^. The injections of Stx2 were performed on the same mice for three times at every 24 h interval. Body weight loss was monitored after LPS and Stx2 administrations; peripheral blood samples were collected at every 24 h interval for analysis.

### Peripheral blood work

Peripheral blood was collected by cardiac puncture with anti-coagulant (0.1 ml of 3.8% sodium citrate per ml of blood). After centrifugation (150 × g, 10 mins, 4 °C), blood was separated into platelet-rich plasma (PRP), white blood cells (WBC) and red blood cells (RBC). PRP was collected followed by further centrifugation (5000 × g, 10 mins, 4 °C) to obtain platelets (PLT) and platelet-free plasma (PFP). WBC were isolated from the buffy coat followed by lysis of RBC using RBC lysis buffer (150 mM NH_4_Cl, 10 mM NaHCO_3_, 0.1 mM EDTA). In some experiments, WBC were further separated into CD11b^+^ and CD11b^−^ WBC sub-populations using a PE-conjugated anti-mouse CD11b antibody (BioLegend) and EasySep™ release mouse PE positive selection kit (STEMCELL Technologies). To assess the severity of HUS, we used several measures: body weight change, a ratio of reticulocytes to erythrocytes in blood, platelet count, blood urea nitrogen levels, and plasma creatinine levels. To determine peripheral reticulocytes, three drops of isolated RBC were mixed with one drop of Reticulocyte Stain (Sigma-Aldrich; 10 mins, room temperature) followed by smearing on a microscope slide and counting of reticulocytes per 1000 RBC. Blood urea nitrogen (BUN) levels were determined by spectrophotometer using a BUN reagent set (Pointe Scientific). Plasma creatinine levels were determined using Creatinine Colorimetric Assay Kit (Cayman Chemical).

### Adoptive transfer

To transfer blood components from LPS-Stx2-induced HUS mice to non-HUS recipients, peripheral blood samples were collected from donor HUS mice 4 h after the 2nd Stx2 administration, followed by separation into WBC, RBC, PLT and PFP. Adoptive transfer was then performed by intravenous (*i.v.*) transfusion of donor PFP (0.3 ml) or cell samples as WBC (1 × 10^6^), RBC (1 × 10^8^) or PLT (5 × 10^8^) in 0.3 ml PBS via the tail vein of recipient mice that had been primed with one dose of LPS (300 μg/kg) 24 h earlier. Blood component transfer from HUS mice to recipients was repeated every 24 h for two-three rounds. Control experiments were performed by transferring blood components from LPS-primed donor mice (no Stx2 administration) or the carrier solution PBS to LPS-primed mice. Prior to and after the adoptive transfer, the recipient mice were monitored for body weight change. The recipient mice were euthanized after the 3rd adoptive transfer, and blood urea nitrogen (BUN), creatinine, peripheral reticulocytes and thrombocytopenia were determined thereafter.

### *Ex vivo* Stx2 treatment

Peripheral WBC, RBC or cells from each fraction of bone marrow (1 × 10^6^ cells each), or PLT (5 × 10^8^) from LPS-primed mice or healthy mice were incubated with Stx2 (10 ng/ml) *ex vivo* in PBS (0.5 ml, 30 min, 25 °C). The cells were then washed thoroughly with PBS (5×) to remove excess Stx2 followed by suspension in PBS (150 µl) and adoptive transfer into LPS-primed recipients (1 × 10^6^ total WBC, 1 × 10^6^ CD11b^+^ or CD11b^−^ leukocytes, 1 × 10^8^ RBC, 5 × 10^8^ PLT, or 1 × 10^6^ bone marrow cells per mouse). Control experiments were done by adoptive transfer of mice with cells that had not been incubated with Stx2 or with the carrier solution PBS.

### Bone marrow (BM) cell isolation and fractionation

Mouse BM was harvested from femur and tibia bones. After lysis of RBC, whole BM cells were fractionated using discontinuous Percoll™ (GE Healthcare) density gradients consisting of layers of 75%, 60%, 50%, and 40% of Percoll density solutions (2 ml per layer) prepared following the sequential decreasing order from the bottom to the top in 15 ml centrifugation tubes. Prepared BM cells in 2 ml PBS were laid on top of the gradients followed by centrifugation at 1800 × g for 40 min (18 °C). Four cell-rich bands were formed at the density interfaces of 0–40%, 40–50%, 50–60% and 60–75% (fractions I, II, III and IV, respectively) after the centrifugation and cells in each band were collected. After washing with PBS to remove excess Percoll, cells in different fractions were determined by flow cytometry for granulocytes (Ly6G^+^), monocytes (Ly6C^high^), myeloid leukocytes (CD11b^+^Gr-1^+^), B lymphocytes (B220^+^), etc. Antibodies with fluorophore conjugations for flow cytometry were obtained from BioLegend.

### Stx2-FITC and toxin-cell binding determination

Fluorescein isothiocyanate (FITC) (Pierce) was chemically conjugated to Stx2 using the EZ-Label FITC Protein Labeling Kit (Thermo Scientific). Briefly, Stx2 (0.1 mg) was incubated with FITC (10 µg) in borate buffer (0.1 ml, pH 8.5) for 2 h (25 °C). The labeling reaction was then quenched with 100 mM Tris buffer (pH 7.5), followed by extensive dialysis against PBS to remove the excess uncoupled FITC. For cell surface labeling, WBC (1 × 10^6^) isolated from LPS-primed mice and unprimed mice were incubated with Stx2-FITC (10 ng/ml) in 0.5 ml PBS for 30 min. After Stx2-FITC labeling, the cells were washed, and labeled with Pacific Blue™-conjugated anti-mouse CD11b, and PE-conjugated anti-mouse Ly6G antibodies (both from BioLegend), followed by flow cytometry analysis.

### Statistical analyses

Data represent at least three independent experiments and are expressed as the mean ± SEM. The reported p value was 2-tailed. Differences were considered statistically significant at *P* < 0.05, as analyzed using the Student *t*-test for paired samples and one-way ANOVA for k > 2 samples.

### Results

**Figure 1:**
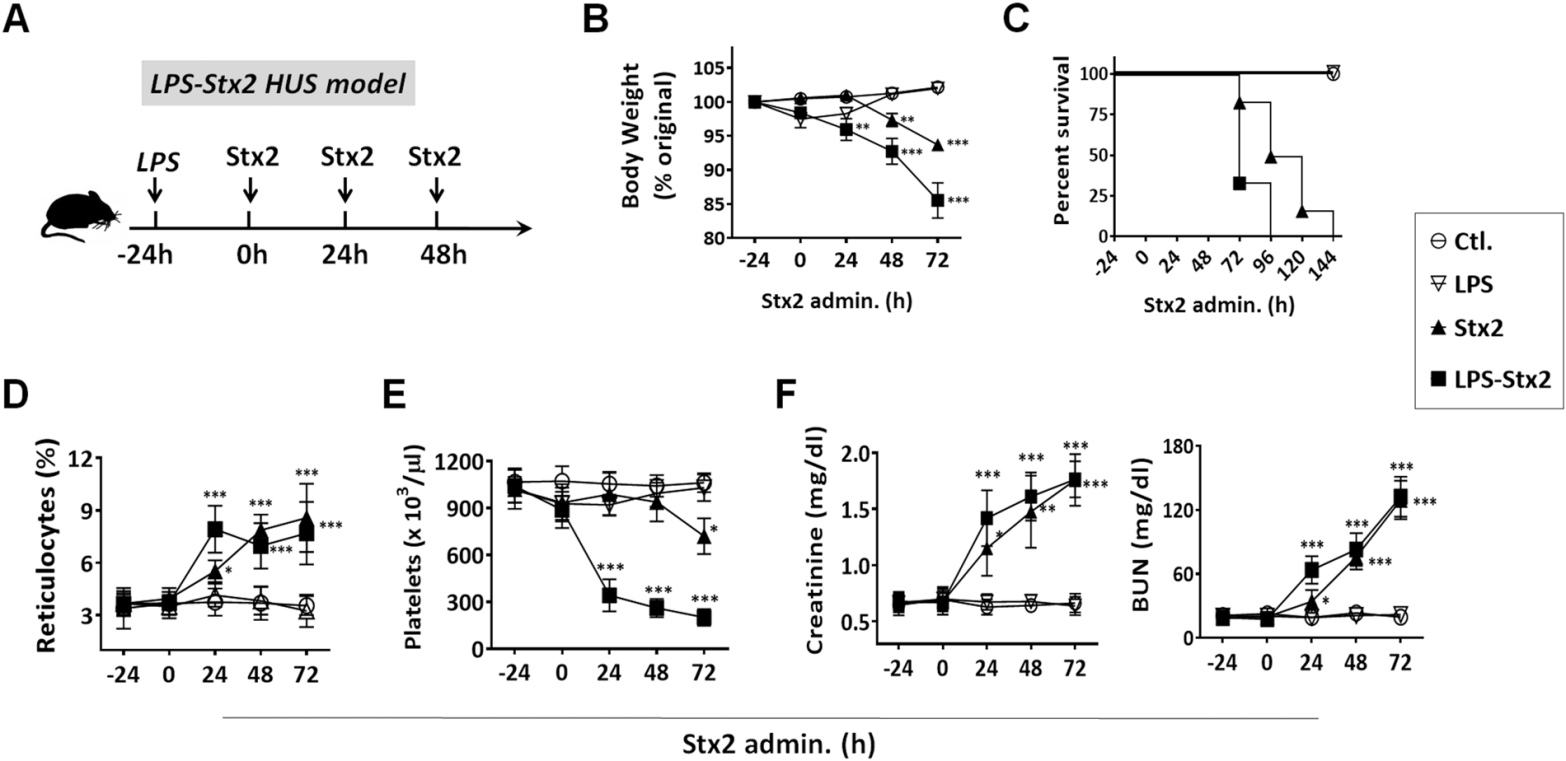
LPS-Stx2 induced HUS in mice. (**A**) The induction scheme. Mice were primed with LPS (300 μg/kg, *i*.*p*.) a day prior to three consecutive Stx administrations (500 ng/kg, *i*.*p*. every 24 h). Controls were mice administered LPS alone or PBS (Ctl.). (**B**–**F**) Determination of HUS. Starting from the LPS treatment, mouse body weight (**B**) was recorded daily. (**C**) Mouse survival rate through the HUS induction. The clinical diagnostic triad including hemolytic anemia, thrombocytopenia and renal dysfunction detected by assaying increases in peripheral reticulocytes (**D**), reduction of peripheral platelets (**E**), and increases in the plasma creatinine and urea nitrogen (BUN) levels (**F**). Data represent over three independent experiments; n = 10, *P < 0.05, **P < 0.01, ***P < 0.001 versus respective controls.

To induce HUS, C57BL/6 mice were first given one dose of LPS (300 μg/kg, i.p.). After 24 h, Stx2 (500 ng/kg) was administered intraperitoneally to the same mice, followed by two additional Stx2 administrations, each given at 24 h intervals (the scheme was shown in Fig. 1A). As shown in Fig. 1(B–F), LPS priming followed by Stx2 administrations (LPS-Stx2) rapidly induced HUS development, as evidenced by body weight loss, thrombocytopenia, anemia as indicated by increased peripheral reticulocytes, and significantly elevated creatinine and BUN levels. Kidney failure was evident after 2 days following the LPS-Stx2 treatments when there was deficit of urinary output. All mice treated with LPS-Stx2 succumbed to HUS in 3–4 days. Comparably, mice primed with LPS alone experienced only transient, slight weight loss without signs of thrombocytopenia, anemia or renal dysfunction. Mice given Stx2 without LPS priming developed similar, but significantly delayed or mild HUS symptoms. For inducing HUS lethality, the mice without LPS pretreatment required higher doses of toxin (1–3 µg/kg) and more frequent treatments (>4 treatments). The mice lacking LPS pretreatment survived for a longer period of time following Stx2 administration than the mice that had been primed prior to Stx2 administration. These results were in agreement with previous studies by others using Stx2-induced HUS mouse models with LPS priming^28,31^.

It has been shown that purified Shiga toxin can elicit proinflammatory cytokines from human and murine WBC^32,33^. To examine the effect of Stx2 on the induction of proinflammatory cytokines, we also assessed the levels of various proinflammatory cytokines in the plasma from mice that developed HUS. As shown in Supplementary Figure 1, the levels of IL-1β, TNF-α, MCP-1, IL-6 and KC were significantly elevated in mice during HUS development.

**Figure 2:**
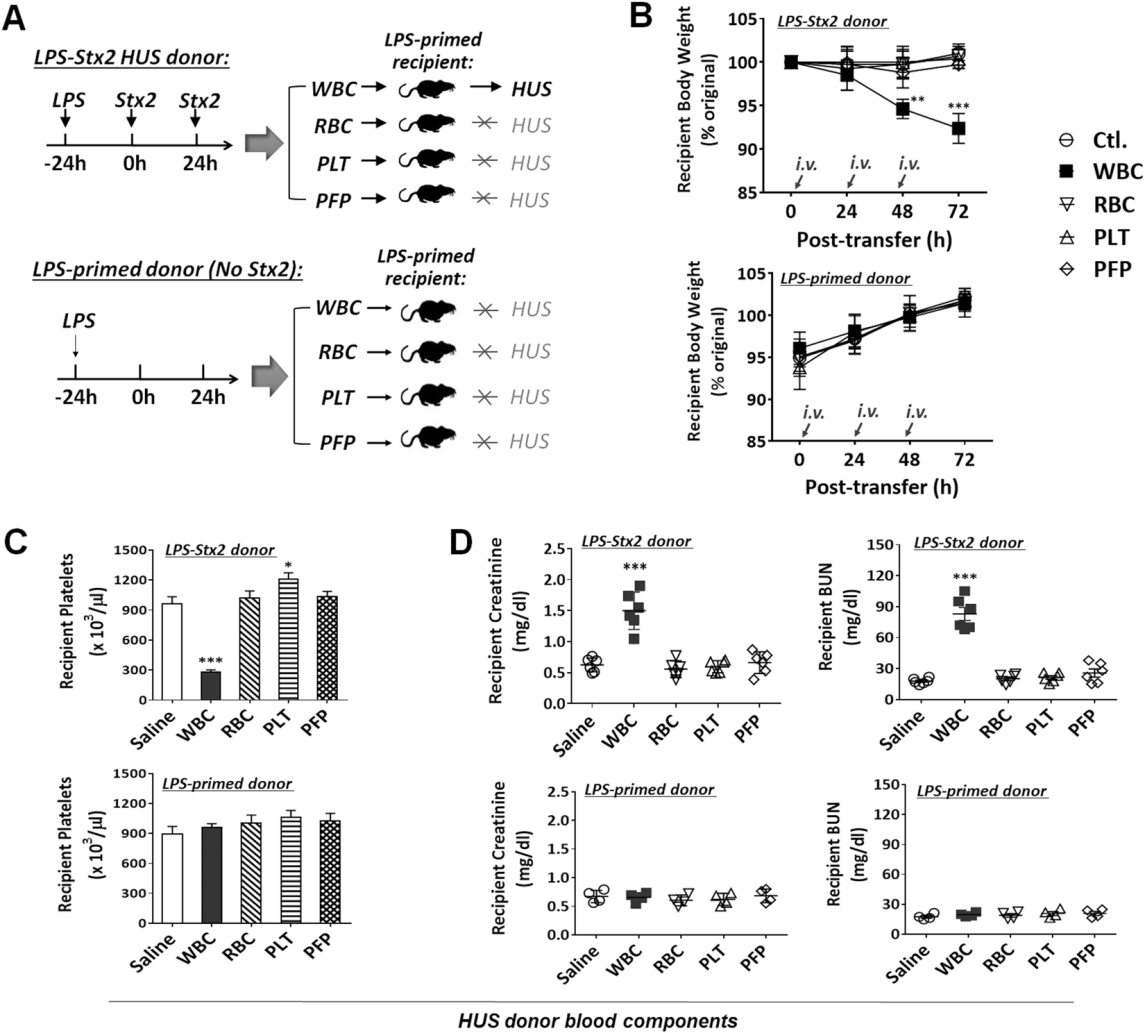
HUS transmission by peripheral white blood cells (WBC) of LPS-Stx2-induced HUS mice. Peripheral blood was obtained from LPS + Stx2-treated (n = 6, experimental group) or LPS-primed (n = 4, control group) mice (donors). The blood components including WBC, RBC, platelets (PLT) and PLT-free plasma (PFP) were separated and transferred intravenously (*i*.*v*., every 24 h) into mice (recipients) that were administered LPS (300 μg/kg, *i*.*p*.) a day earlier (priming). (**A**) The experimental scheme; (**B**–**D**) HUS development in recipient mice. Data show the body weight loss (**B**), and the appearance of thrombocytopenia (**C**) and renal dysfunction indicated by elevated plasma creatinine and urea nitrogen (BUN) levels (**D**) 72 h after the adoptive transfer. Data represent four independent experiments; *P < 0.05, **P < 0.01, ***P < 0.001 versus respective controls.

As Stx2 was given intraperitoneally, the toxin was required to be transported to the kidneys in order to cause damage. To determine if circulating blood cells played a role in carrying Stx2 to the kidneys, peripheral blood samples were collected from LPS-primed mice 4 h after the 2^nd^ Stx2 administration, during which HUS was developing. These HUS blood samples were separated into platelet-free plasma (PFP), platelets (PLT), RBC and WBC by differential centrifugations, followed by adoptive transfer (*i*.*v*.) of each component into recipient mice that were similarly primed by LPS but without Stx2 administration (Fig. 2A). A total of three rounds of blood component transfer were performed in every 24 h interval. To examine the effect of LPS on HUS development in Stx2-treated mice, the peripheral blood components were also isolated from mice primed by LPS but without Stx2 treatment, and then adoptive transferred into recipient mice (Fig. 2A). As shown in Fig. 2(B–D), recipient mice, although never having been directly exposed to Stx2, developed HUS after receiving WBC from LPS-Stx2 donors. The WBC recipient mice demonstrated characteristic HUS symptoms including body weight loss (Fig. 2B), thrombocytopenia (Fig. 2C) and renal dysfunction (Fig. 2D). Indeed, these transferred WBC caused HUS in recipient mice similar to that of HUS as induced by LPS-Stx2. In contrast, mice that received PFP, PLT or RBC from mice with HUS failed to develop HUS. As expected, recipient mice that received WBC from mice that were primed by LPS, but not treated with Stx2, did not develop HUS. In conclusion, these results showed that WBC from HUS*-*inflicted mice were capable of transferring the toxin-mediated effect to non-HUS mice, suggesting that WBC serve as the Stx2 carrier to distal target organs. Other blood components such as PFP, PLT or RBC were incapable of inducing HUS in recipient mice.

**Figure 3:**
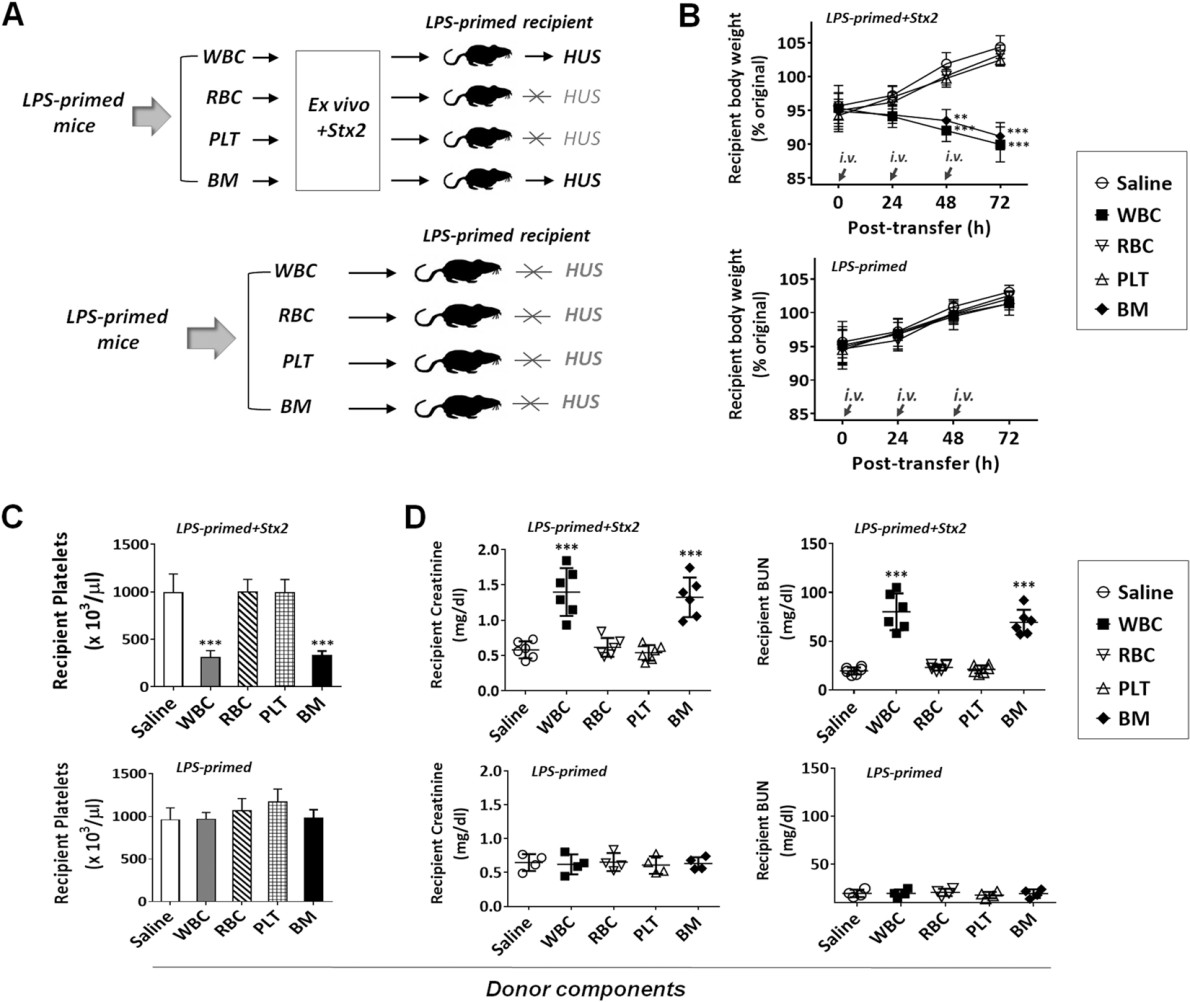
*Ex vivo* exposure of WBC to Stx2 confers capacity to cause HUS in recipient mice. Peripheral blood WBC, RBC and PLT, as well as bone marrow (BM) cells, were isolated from LPS-primed mice without HUS. The cells were then incubated *ex vivo* with Stx2 (10 ng/ml in PBS, experimental group) or saline (PBS, control group) for 30 min (25 °C), followed by thorough wash and transfer (*i*.*v*.) into other LPS-primed recipient mice (n = 6 for experimental group, n = 4 for control group). (**A**) The experimental scheme. (**B–D**) HUS development in recipient mice assessed by body weight loss (**B**), thrombocytopenia (**C**) and renal dysfunction (**D**). Data represent four individual experiments; *P < 0.05, **P < 0.01, ***P < 0.001 versus respective controls.

To further confirm that WBC can carry Stx2 to kidneys resulting in renal damage, we isolated WBC from LPS-primed, non-Stx2-exposed mice and incubated these WBC with or without Stx2. In these experiments, WBC, as well as other types of cells, isolated from the peripheral blood or bone marrow of LPS-primed mice were incubated with or without Stx2 (10 ng/ml, ~2 CD50 for Vero cells) *ex vivo* for 30 min (25 °C), followed by thorough washing prior to transfer into recipient mice (Fig. 3A). As shown in Fig. 3(B–D), Stx2-treated WBC and BM cells rapidly induced HUS in recipient mice, suggesting that WBC bound Stx2 during the *ex vivo* exposure and carried the toxin *in vivo* to cause the disease. Conversely, *ex vivo* exposure to Stx2 did not enable RBC or platelets to cause HUS. In agreement with results in Fig. 2, WBC and BM cells isolated from mice with LPS priming but without Stx2 treatment did not induce HUS in recipient mice.

**Figure 4:**
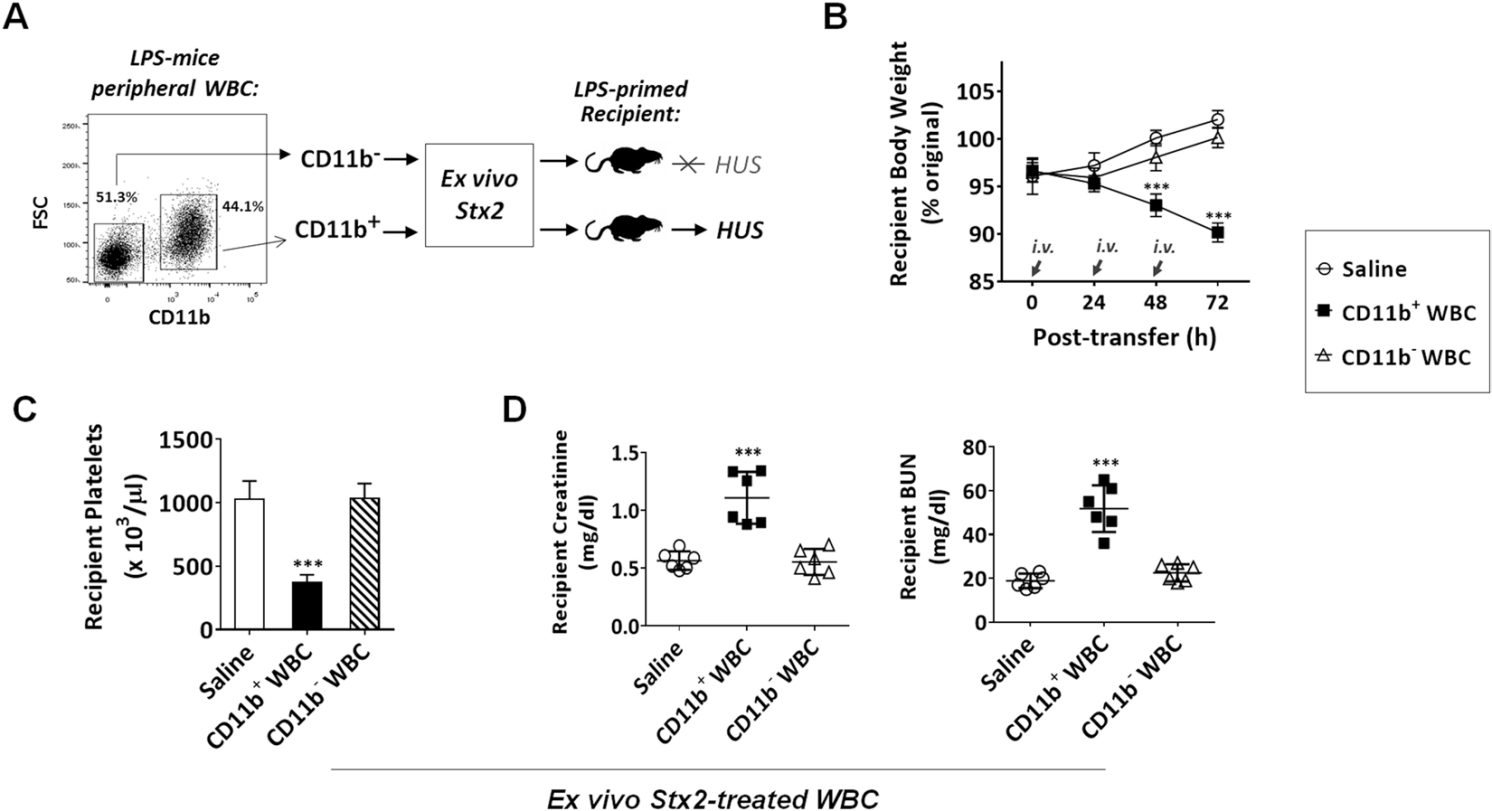
CD11b^+^ myeloid leukocytes carry Stx2 and cause HUS *in vivo*. Peripheral WBC from LPS-primed, non-HUS mice were further separated into CD11b^+^ and CD11b^−^ leukocyte populations by antibody-facilitated positive selection. After brief incubation (30 min) with Stx2 (10 ng/ml in PBS) *ex vivo* and thorough washing, two populations of leukocytes were transferred (*i*.*v*.) into different, LPS-primed recipient mice (**A**). HUS development in recipient mice assessed by body weight loss (**B**), thrombocytopenia (**C**) and renal dysfunction (**D**). Data represent two individual experiments; n = 6; *P < 0.05, **P < 0.01, ***P < 0.001 versus respective controls.

Since WBC are heterogeneous, containing both CD11b^+^ myeloid leukocytes and CD11b^−^ WBC (Fig. 4A), we separated these populations by antibody-mediated positive selection using magnetic beads. Subsequent exposure of CD11b^+^ and CD11b^−^ cells to Stx2 *ex vivo*, and then testing for capability of causing HUS *in vivo*, demonstrated that only CD11b^+^ myeloid leukocytes carried Stx2 leading to HUS. As shown in Fig. 4(A–D), CD11b^+^ leukocytes, but not CD11b^−^ WBC, effectively induced HUS in recipient mice.

**Figure 5:**
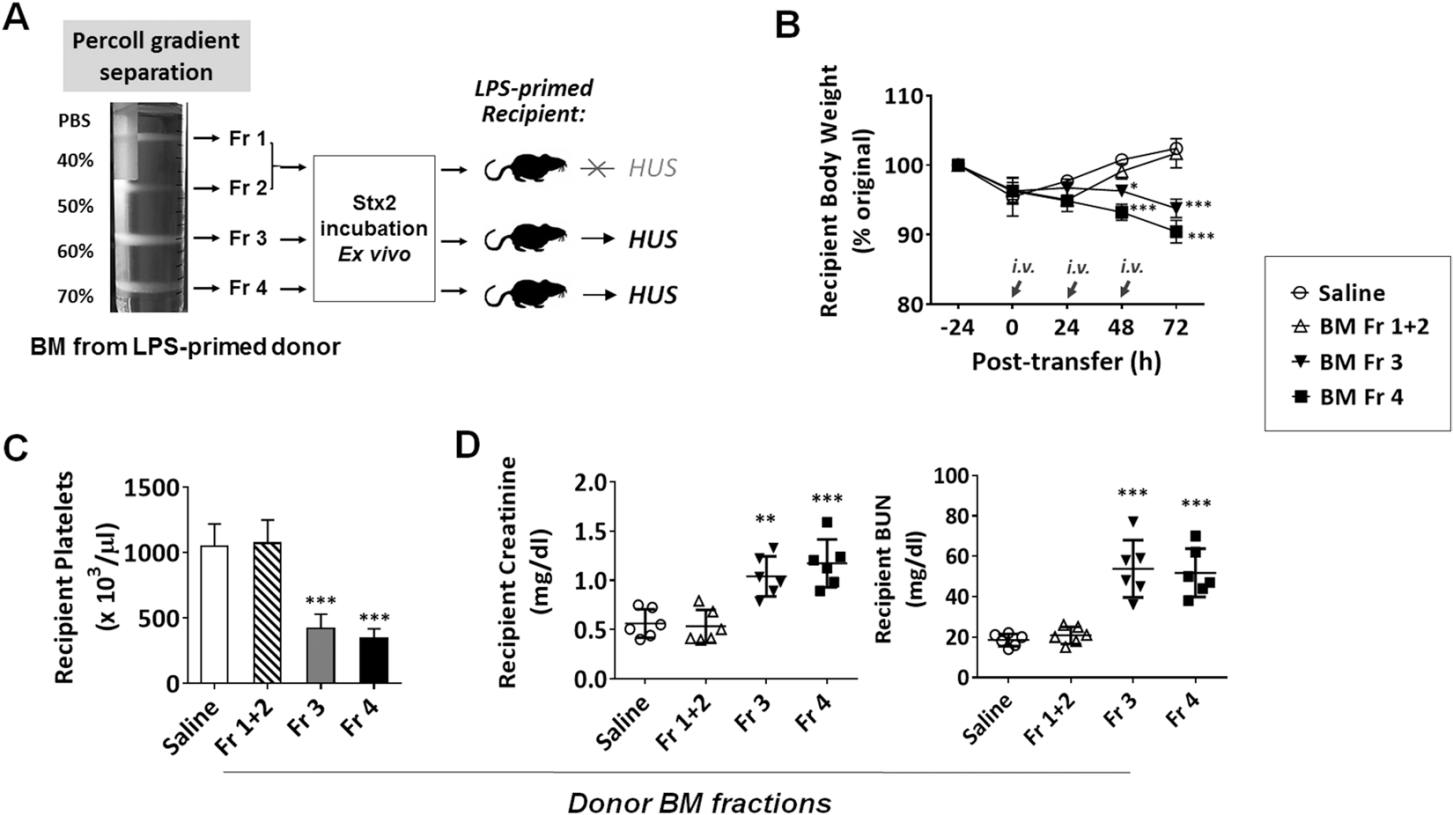
Myeloid leukocytes from BM also have the capability to carry Stx2. Total BM cells from LPS-primed mice without HUS were fractionated by Percoll density gradients into non-myeloid cells (Fr. I + II) and myeloid leukocytes (Fr. III and Fr. IV) following a protocol described previously. (**A**) The separated populations were incubated with Stx2 (10 ng/ml in PBS) *ex vivo*, followed by thorough washing and transfer into other LPS-primed mice (recipients). (**B**–**D**) HUS development in recipient mice assessed by body weight loss (**B**), thrombocytopenia (**C**) and renal dysfunction (**D**). Data represent two individual experiments; n = 6; *P < 0.05, **P < 0.01, ***P < 0.001 versus respective controls.

To further identify which WBC were the carriers for Stx2, we isolated cell types using another procedure. We separated BM cells by Percoll density gradients. As shown in Fig. 5A, CD11b^+^ myeloid leukocytes were enriched in the high-density fractions between the Percoll densities of 50–60% and 60–70% (fractions III and IV). The CD11b^+^ fraction III contained Ly6C^high^ monocytes and immature Ly6G^+^ granulocytes (approximately 40% and 60%, respectively), while the highest density fraction IV contained those CD11b^+^ that were nearly all mature Ly6G^+^ granulocytes (>95%). Low density fractions I and II chiefly contained CD11b^−^ lymphocytes, progenitors of leukocytes and stroma cells. After cell separation and washing, different fractions of BM leukocytes were exposed to Stx2 *ex vivo* by co-incubation, prior to transfer into recipient mice (Fig. 5A). As shown in Fig. 5(B–D), fraction IV granulocytes carrying Stx2 caused the most severe HUS in recipients, while the fraction III leukocytes induced relatively mild, delayed HUS symptoms. Conversely, BM cells from the low-density fractions (I and II) failed to induce HUS *in vivo*. In conclusion, these results collectively support that CD11b^+^ leukocytes serve as the Stx2 carrier during HUS development.

**Figure 6:**
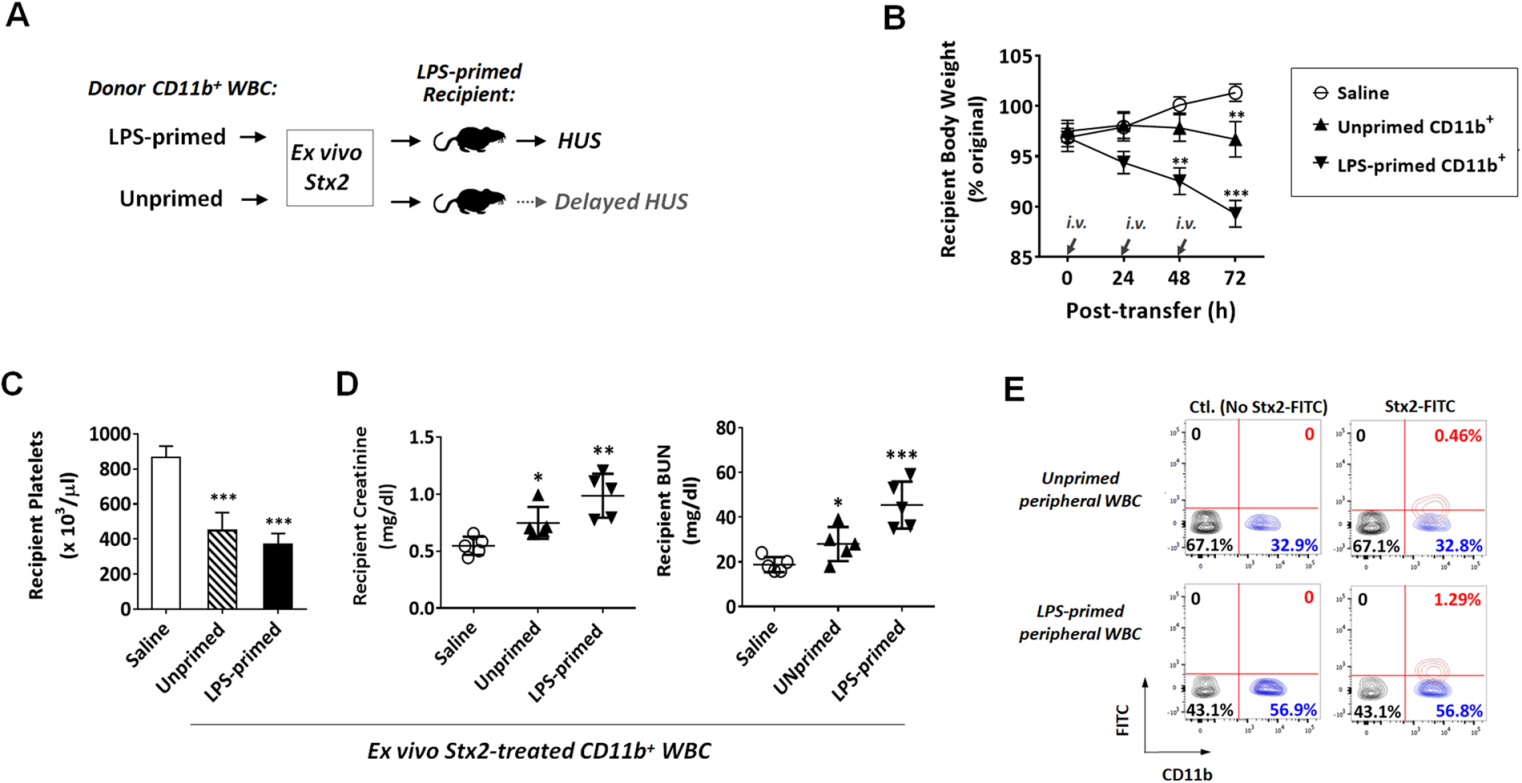
LPS priming promotes CD11b^+^ leukocytes binding to Stx2. CD11b^+^ leukocytes isolated from LPS-primed and unprimed donors were incubated with Stx2 (10 ng/ml in PBS) *ex vivo*, followed by washing and transfer into LPS-primed recipient mice. (**A**) Experimental scheme. (**B–D**) HUS development in recipient mice assessed by body weight loss (**B**), thrombocytopenia (**C**) and renal dysfunction (**D)**. (**E**) Increased population of Stx2-FITC-binding CD11b^+^ leukocytes from LPS-primed mice than those from unprimed mice, as analyzed by flow cytometry. Data represent two individual experiments; n = 5; *P < 0.05, **P < 0.01, ***P < 0.001 versus respective controls.

Interestingly, we also found that, given equivalent *ex vivo* Stx2 exposure and equivalent amounts of leukocytes being transferred, CD11b^+^ leukocytes isolated from LPS-primed mice and mice without LPS priming displayed differential potency in inducing HUS. As shown in Fig. 6(A–D), after co-incubation with Stx2, CD11b^+^ leukocytes from LPS-primed mice exhibited a much stronger capability to induce HUS than those from healthy mice, which had not been LPS-primed; after the adoptive transfer, recipient mice with the former leukocytes demonstrated faster body weight loss and more profound renal dysfunction than mice with the latter. These results suggest that LPS priming significantly increased the capacity for CD11b^+^ leukocytes to bind and/or to carry Stx2, whereas leukocytes without LPS priming only had a weak capacity to deliver the toxin. Binding of CD11b^+^ leukocytes with FITC-labeled Stx2 confirmed this finding, showing a 3~5-fold increase in the Stx2-positive population of leukocytes from LPS-primed mice compared to that from unprimed mice (Fig. 6E, Table 1). Further labeling of CD11b^+^ leukocytes with anti-Ly6G antibodies demonstrated that Stx2-bound leukocytes were a small group of CD11b^+^Ly6G^−^ cells. We also compared the effect of LPS-primed, Stx2-bound WBC on the development of HUS in LPS-primed recipients versus that in unprimed recipients. In this experiment, WBC were isolated from LPS-primed mice, incubated with Stx2 and then adoptively transferred into LPS-primed or unprimed recipient mice (Supplementary Figure 2A). As shown in Supplementary Figure 2(B–D), LPS-primed, Stx2-bound CD11b^+^ leukocytes exhibited a stronger capability of inducing HUS in LPS-primed recipients than in recipients without LPS priming; after the adoptive transfer, LPS-primed recipients demonstrated faster body weight loss and more profound renal dysfunction than unprimed mice. Taken together, our results suggest that LPS-induced systemic inflammation plays a significant role in promoting HUS; it primes CD11b^+^ leukocytes for greater binding to Stx2, rendering these leukocytes more effective disseminators of Stx2 to induce HUS.

**Table 1:** Cell count for each subtype of peripheral WBC.

Cell Count (/µl)	CTL				Stx2-FITC			
	CD11b^−^		CD11b^+^		CD11b^−^		CD11b^+^	
	Stx2^−^	Stx2^+^	Stx2^−^	Stx2^+^	Stx2^−^	Stx2^+^	Stx2^−^	Stx2^+^
Unprimed peripheral WBC	6015 ± 490	0	2950 ± 282	0	5903 ± 514	0	2877 ± 314	66 ± 22
LPS^−^primed peripheral WBC	5936 ± 796	0	5172 ± 770***	0	5792 ± 692	0	5258 ± 798***	208 ± 25**

**P < 0.01, ***P < 0.001 versus the same subtype in unprimed peripheral WBC.

**Figure 7:**
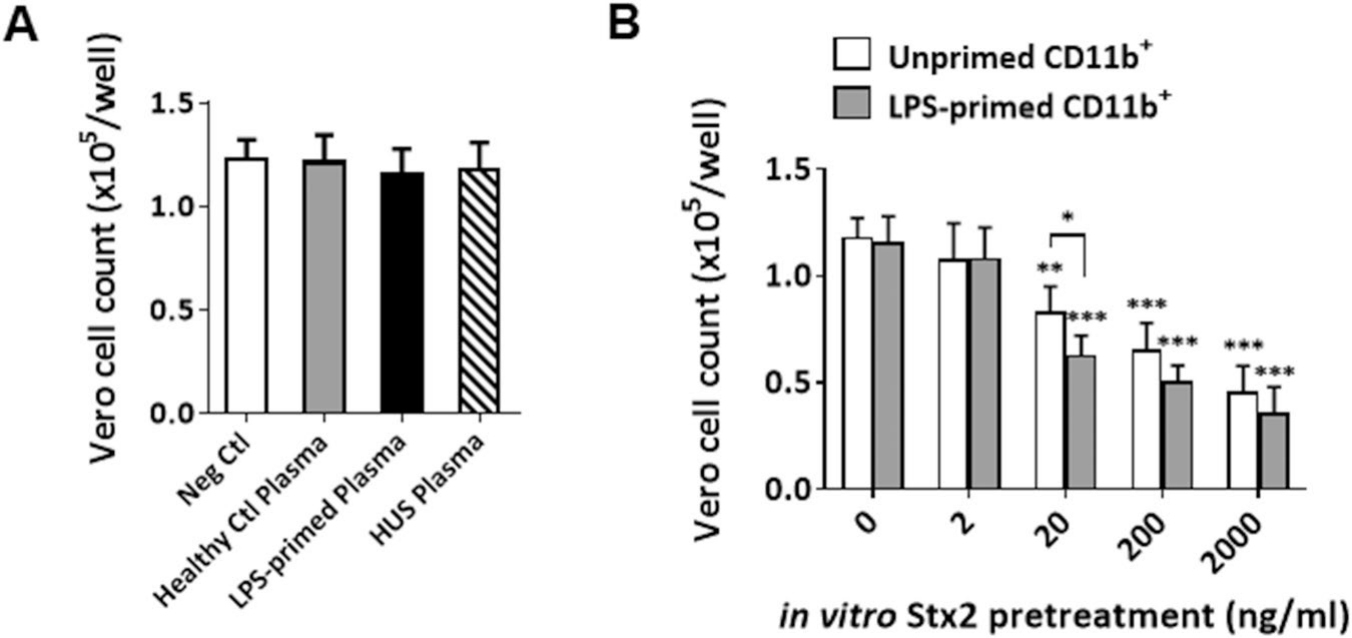
CD11b^+^ leukocytes carry and deliver Stx2 to Vero cells. Vero cells labeled with CSFE were cultured in a 96-well plate (~1 × 10^5^ cells/well) for 12 h. Excessive dye was washed off prior to the culture. Cell viability after the treatment was determined by counting cells under a fluorescent microscope. (**A**) Plasma toxicity study. Plasma were obtained from healthy control mice, LPS-primed mice (300 μg/kg, i.p; 48 h after the injection) or HUS-developed mice (4 h after 2^nd^ Stx2 i.p. injection, as described in manuscript), followed by the incubation with Vero cells (100 μl each well, 37 °C, 24 h). (**B**) Stx2 delivery study. CD11b^+^ cells isolated from the peripheral blood of LPS-primed or unprimed mice were incubated with a series of concentrations of Stx2 (0, 2, 20, 200 and 2000 ng/ml, respectively; 25 °C, 30 min). After thoroughly washing, Stx2-treated CD11b^+^ cells were incubated with Vero cells (1 × 10^5^ CD11b^+^ cells each well, 37 °C, 24 h). Data represent three independent experiments; n = 4; *P < 0.05, **P < 0.01, ***P < 0.001 versus respective controls.

Finally, to illustrate that CD11b^+^ leukocytes are capable of carrying and delivering Stx2 to renal endothelial cells, we studied the interactions between Vero cells (kindly provided by Dr. Margo Brinton, Georgia State University) and various mouse CD11b^+^ leukocytes and plasma samples *in vitro*. The CD50 of Stx2 for Vero cells was determined to be 5.68 ng/ml according to the method previously described^34^. In this experiment, plasma samples were isolated from healthy control mice, LPS-primed mice and HUS-developed mice. CD11b^+^ leukocytes were isolated from LPS-primed and unprimed mice, then incubated with or without Stx2 at different concentrations. After thoroughly washing off unbound Stx2, CD11b^+^ leukocytes or plasma were incubated with Vero cells. As shown in Fig. 7A, all plasma samples had no effect on the viability of Vero cells, suggesting that plasma samples contain no pathogenic concentration of Stx2. In contrast, after incubation with Stx2, CD11b^+^ leukocytes conferred Vero cell death (Fig. 7B). In agreement with the previous result that LPS facilitates Stx2-CD11b^+^ leukocyte binding, CD11b^+^ leukocytes primed by LPS tended to induce a higher amount of Vero cell apoptosis than unprimed CD11b^+^ leukocytes did. The results suggest that CD11b^+^ leukocytes, particularly LPS-primed CD11b^+^ leukocytes, are able to effectively carry and deliver Stx2 to renal endothelial cells.

The binding of Stx2 to CD11b^+^ leukocytes was further characterized via two control experiments (Supplementary Figure 3). First, CD11b^+^ leukocytes were incubated with Stx2-FITC (10 ng/ml), as well as unlabeled Stx2 (from 0 to 2000 ng/ml) for the competitive binding. Second, antibody against Gb3 (from 0 to 10 μg/ml) and Stx2-FITC (10 ng/ml) were co-incubated with CD11b^+^ leukocytes. The binding of Stx2-FITC to CD11b^+^ leukocytes was determined by flow cytometry. As shown in Supplementary Figure 3A, a high concentration of unlabeled Stx2 almost completely removed the binding of Stx2-FITC to CD11b^+^ leukocytes, suggesting that the binding of Stx2 to CD11b^+^ leukocytes may be mediated by a specific receptor. The binding assay also showed that anti-Gb3 antibody did not affect Stx2-CD11b^+^ leukocyte binding (Supplementary Figure 3B), suggesting that the receptor mediating Stx2-CD11b^+^ leukocyte binding is not Gb3.

## Discussion

Due to the broad sources of STEC transmission such as consumption of contaminated food and water, or direct contact with infected persons or animals, outbreaks of STEC occur frequently each year causing millions of illnesses. While STEC infection is restricted to the intestines, patients, especially children and elders, are at risk for developing HUS – a severe, life-threatening condition occurring when bacteria-associated Stx the enters and damages renal cells. Approximately 15% of STEC-infected patients develop HUS in clinics, and acute kidney failure is among the most common reasons leading to lethality in STEC-infected individuals. STEC produces two types of bipartite toxins, Stx1 and Stx2, both of which contain an A subunit and five B subunits (AB_5_). The B subunits mediate cell attachment through binding to cell surface receptors, while the A subunit possesses enzymatic activity that cleaves a single adenine residue from a prominent stem-loop structure that makes up part of the peptidyltransferase reaction center of 28 S rRNA, resulting in ribosome and gene damage^6,35–37^. A few cell types including renal epithelial cells express the high affinity receptors for the B subunits, rendering kidneys vulnerable to the entry of Stx^12,38,39^. However, STEC are non-invasive bacteria restricted to the intestines. To cause renal damage, STEC-produced Stx must be transported from the intestines to the kidneys. Identifying the vehicle delivering Stx from the site of infection to the target distal organs might illuminate mechanisms for predicting and/or preventing subsequent HUS.

The establishment of procedures for reliable Stx-induced HUS in mice is an important step in research. Initial studies of these procedures indicated that, in addition to Stx administration, prior systemic LPS administration allows for more reliable induction of HUS that mimics pathological manifestations in human disease^28,31^. Mere administration of Stx2 induces partial symptoms of HUS which are also less severe^28^. In our study, we utilized the LPS-Stx2 procedure for inducing HUS in mice^28,29,31^. The objective of our experiments was to identify the carriers of Stx2 to the kidneys where the toxin results in renal damage/failure. Four sequential experiments were performed in this investigation and collectively led to the conclusion that CD11b^+^ myeloid leukocytes bind Stx2 and act as the exclusive toxin carriers to the kidneys, resulting in lethal HUS *in vivo*. In our first set of experiments, we harvested peripheral blood cells and the cell-free plasma from LPS-Stx2 treated mice in which severe HUS was developing, and transferred these components to recipient mice that had no prior Stx2 exposure. These experiments showed that peripheral WBC, but not other cells from mice with HUS, carried toxin and transferred the disease to recipients. Cell-free plasma from the same HUS mice also failed to transfer the disease. Because cell-free plasma did not exhibit the capacity to induce HUS, our results rule against extracellular microvesicles^27^ being the primary modality for disseminating the toxin.

In our second approach, we tested whether circulating blood cells from mice without HUS that were briefly exposed to Stx2 *ex vivo* could cause HUS *in vivo* after adoptive transfer into recipients. The results showed that WBC, as well as myeloid-rich BM cells, gained the capability to induce HUS after exposure to Stx2 *ex vivo*. These findings suggest that Stx2 could bind to these cells *ex vivo* and be carried by them in the circulation of live animals. In our third approach, we further separated peripheral WBC into CD11b^+^ myeloid leukocytes and CD11b^−^ WBC, and showed that the former leukocytes serve as the Stx2 carrier. Brief exposure to Stx2 *ex vivo* was sufficient for CD11b^+^ leukocytes to cause HUS *in vivo*. In all the “*trans*”-induced HUS experiments performed by adoptive transfer of Stx2-exposed leukocytes, renal dysfunction was evident, suggesting the Stx2-bearing leukocytes were carrying toxin to the kidneys via blood circulation. Lastly, we tested leukocytes harvested from mice which had or had not been primed with LPS. We showed that those leukocytes harvested from LPS-primed mice had a higher number of cells capturing Stx2 *ex vivo* and induced more profound HUS *in vivo*.

We showed that LPS priming in the donor and the recipient mice, prior to the adoptive transfer of *ex-vivo* Stx2 acquired CD11b^+^ cells, is critical to induce severe HUS with the complete clinical triad. The LPS-priming is certainly consistent with the protocol for inducing HUS in mice, which entails peritoneal exposure to both LPS and Stx2^28,29,31,40,41^. In agreement with previous studies that the pre-treatment of mice with LPS renders the animals more susceptible to the development of signs and symptoms of HUS following intraperitoneal (i.p.) administration of Stx^28,31,40^, our results also showed an increased number and Stx2-binding capability of Stx2^+^ leukocytes harvested from LPS-primed mice (Fig. 6E, Table 1). The LPS-primed primed leukocytes thus effectively delivered Stx2 to the target organs through the blood circulation that caused HUS in recipients. This finding suggests that LPS, possibly through inflammatory cytokine exposure or directly inducing intracellular signaling, may prompt expression of receptors that are responsible for carrying Stx2. This finding is also in agreement with the previous study by Clayton and co-workers showing that LPS can upregulate Stx receptors in a primate model of HUS^42^. Studies of intestinal inflammation^43,44^ have shown that innate leukocytes change their protein expressions under the given inflammation. An extended time requirement for leukocytes to express potential receptors for carrying Stx2 following exposure to inflammatory conditions is consistent with the “window period” between the debut of severe enteritis attributable to STEC and the emergence of HUS in the clinic. It is also worthy to note that a previous study by Lentz *et al*.^45^ had shown that early administration of TNF-α could block Stx1-induced disease progression, while TNF-α production after intoxication significantly exacerbates disease. As our results also found that the level of TNF-α, as well as other proinflammatory cytokines, was elevated in mice during HUS development (Supplementary Figure 1), LPS priming may facilitate HUS development in mice through promoting the production of proinflammatory cytokines such as TNF-α. Although not investigated here, it is possible that the priming by systemic inflammation also changes the local environment in the kidney, rendering the tissue more vulnerable to dissemination of Stx2 and more vulnerable to the damaging impact of the toxin. Investigation of how inflammation converges with Stx2 to induce HUS has the potential for yielding better diagnostics and therapeutics for STEC-associated HUS.

## Electronic supplementary material

Supplementary Figures
