# Supplementary material for: LPS-primed CD11b+ leukocytes serve as an effective carrier of Shiga toxin 2 to cause hemolytic uremic syndrome in mice
Source: Sci Rep. 2018 Mar 5;8:3994. doi: 10.1038/s41598-018-22327-4 (PMC5838166; doi:10.1038/s41598-018-22327-4)
Supplement: Supplementary file 1 — Supplementary Figures [file 41598_2018_22327_MOESM1_ESM.docx]

**LPS-primed** **CD11b^+^ leukocytes serve as an effective carrier of Shiga toxin 2 to cause hemolytic uremic syndrome in mice**

Shuo Niu, John Paluszynski, Zhen Bian, Lei Shi, Koby Kidder and Yuan Liu^*^

**Figure list**

Supplementary Figure 1

Supplementary Figure 2

Supplementary Figure 3





Supplementary Figure 1. Proinflammatory cytokine levels in the plasma of mice during HUS development. Cytokines were measured using LEGENDplex™ (BioLegend) according to the protocol provided by the manufacturer. Data represent two individual experiments with n=4; **P < 0.01, ***P < 0.001 versus healthy controls (represented as -24 h time point in the figure).


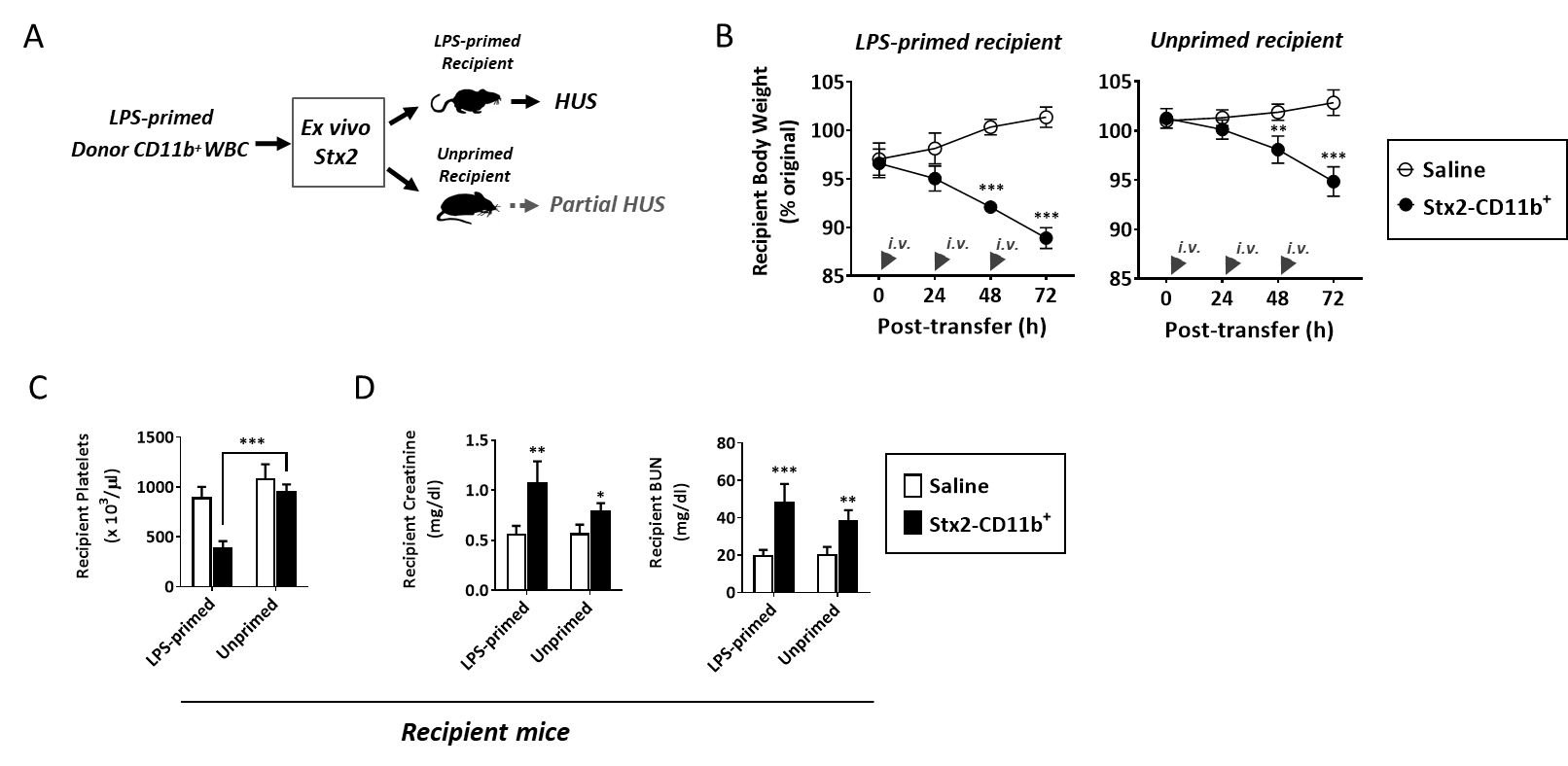


Supplementary Figure 2. Recipient mice without LPS priming developed partial HUS after receiving LPS-primed, Stx2-carrying CD11b^+^ WBC. In the experiment, CD11b^+^ WBC were isolated from LPS-primed donors and then were incubated with Stx2 (20 ng/ml in PBS) *ex vivo*. After washing off unbound Stx2, cells were transferred into LPS-primed or unprimed recipient mice. A) Experimental scheme. B-D) HUS development in recipient mice assessed by body weight loss (B), thrombocytopenia (C) and renal dysfunction (D). Data represent three individual experiments with n=4; *P < 0.05, **P < 0.01, ***P < 0.001 versus respective controls.


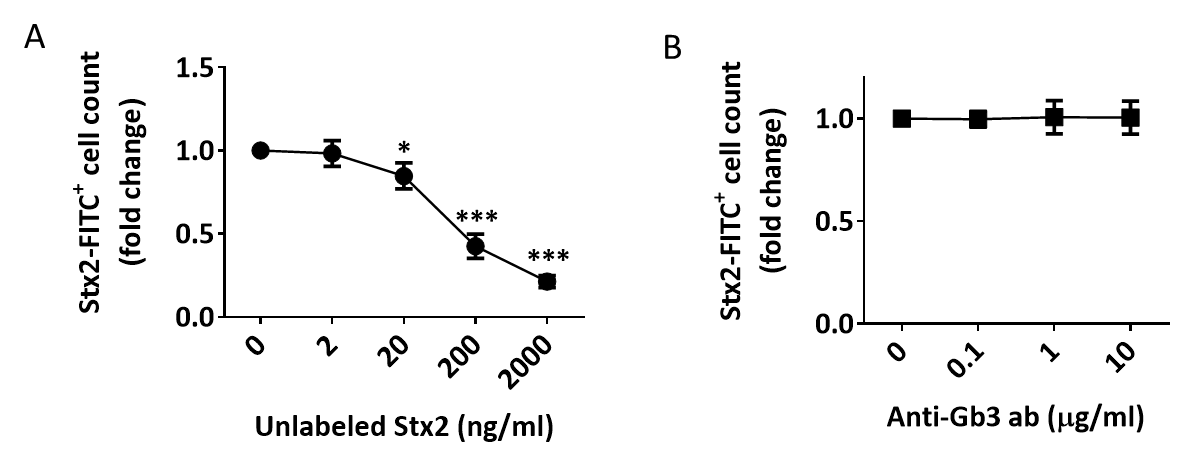


Supplementary Figure 3. Characterization of Stx2-CD11b^+^ leukocyte binding. CD11b^+^ leukocytes were isolated from LPS-primed mice and then incubated with Stx2-FITC (20 ng/ml). The binding of FITC-Stx2 to CD11b^+^ leukocytes was then challenged by unlabeled Stx2 (0, 20, 200, 2000 ng/ml, respectively) (A) or anti-Gb3 antibodies (0, 0.1, 1, 10 μg/ml, respectively) (B) at room temperature for 30 min. Stx2-FITC positive cells were then determined by flow cytometry. Data represent three individual experiments with n=4; **P < 0.01, ***P < 0.001 versus respective controls.
